# The prevalence of orthorexia in exercising populations: a systematic review and meta-analysis

**DOI:** 10.1186/s40337-023-00739-6

**Published:** 2023-02-06

**Authors:** Stine Marie Hafstad, Jonas Bauer, Anette Harris, Ståle Pallesen

**Affiliations:** 1 Department of Psychosocial Science, University of Bergen, Post Box 7807, 5020 Bergen, Norway; 2 Optentia, the Vaal Triangle Campus of the North-West University, Vanderbijlpark, South Africa

**Keywords:** Review, Meta-regression, Meta-analysis, Orthorexia, Prevalence, Frequency, Exercise, Eating disorder

## Abstract

**Aim:**

Orthorexia Nervosa (ON) describes a pathological obsession with proper and high-quality nutrition that is necessary to research further in order to elucidate its prevalence and correlates which may bear implications for prevention and treatment. The aim of this study was to review studies that report the prevalence of ON in people who exercise, calculate an overall prevalence through a random-effects meta-analysis approach and investigate the association of ON prevalence using a random-effects meta-regression. In addition, a sub-group-analysis based on ON-instruments and a sensitivity analysis excluding students samples, were conducted.

**Method:**

Systematic searches were conducted in the following online databases: PubMed, Embase, Web of Science, PsychInfo, CINAHL, Google Scholar and OpenNet. The following search terms were used: Orthore* AND (prevalenc* OR incidenc* OR frequen* OR cut-off OR epidem*). A total of 613 unique hits were reviewed by two blinded authors, and 24 studies were coded and assessed for risk of bias (Holy et.al). The meta-regression included three independent variables (sex, type of sport, and sample size).

**Results:**

The overall prevalence of ON in the exercising population was 55.3% (95% CI 43.2–66.8). Cochran’s *Q* was 11,436.38 (*df* = 23, *p* < 0.0000), and the *I*^2^ was 98.4%, indicating high heterogeneity across studies. The sensitivity showed an overall prevalence of 51.3% (95% CI 51.3–70.0). There was a significant difference in prevalence estimates based on the instruments used (Q_bet_ = 33.6, *df* = 2, *p* < 0.01).

**Discussion:**

The overall prevalence of ON in exercising populations was very high. The between-study disparity was large and was partly explained by the ON-instrument administered. One fourth of the studies had a moderate risk of bias. The majority of the studies did not specify relevant demographic information about the sample, and information about the type of sport was frequently missing.

**Supplementary Information:**

The online version contains supplementary material available at 10.1186/s40337-023-00739-6.

## Plain English summary

Orthorexia Nervosa (ON) describes a pathological obsession with proper and high quality nutrition that is characterized by a restrictive diet, ritualized patterns of eating, and rigid avoidance of foods believed to be unhealthy or impure. This obsession is found among sports athletes in some studies, and there is an assumed link between ON and exercise in general. In this study, the term exercise is defined as any activity requiring physical effort, carried out to sustain or improve health and fitness. In this systematic review and meta-analysis, we examined the overall prevalence of ON in the exercising population. Searching in scientific databases resulted in 613 articles, of which 24 met the rigorous inclusion criteria. The overall prevalence of ON in the general exercising population was 55.3%. The prevalence was thought to be predictable by sex, type of sport, and sample size, but no significant associations were found. This may be due to poor study quality and a lack of demographic information on the participants in some of the included studies. Other predictors worth investigating might be age, student status, status as vegetarian/vegan, psychiatric comorbidity, and intensity/frequency of exercise. This knowledge may bear implications for the prevention and right treatment for people at risk of developing ON.

**Supplementary Information:**

The online version contains supplementary material available at 10.1186/s40337-023-00739-6.

## Introduction

Orthorexia Nervosa (ON), describes an obsession with proper and high-quality nutrition that is characterized by a restrictive diet, ritualized patterns of eating, and rigid avoidance of foods believed to be unhealthy or impure. ON, which literally means “correct appetite”, is a relatively new syndrome, which some researchers regard as a type of eating disorder. Currently, no consensus has been reached with respect to diagnosis categorization [1]. ON was named by Steven Bratman in 1997 [2]. He observed that some people became obsessive and dysfunctional in their way of trying to eat a perfect diet. In the book *Health Food Junkies* [3], he describes this phenomenon in detail. Orthorexics typically restrict their food consumption according to what they believe is pure and healthy because their main motivation is to achieve “optimal health” [4].

Orthorexics overvalue the ideas regarding the health benefits of their diet. However, the food restrictions and rigid avoidance of unhealthy food implied, are considered to be unhealthy. It may lead to nutritional deficiencies, malnourishment, and unwanted/unhealthy weight loss [5, 6]. Psychosocial consequences occur as well, e.g., marked psychological distress [7]. Hence, there is an ongoing discussion regarding ON pertains to an eating disorder (ED) or obsessive–compulsive disorder (OCD) spectrum [8].

According to Cena’s review [9] it’s concluded that ON and EDs imply same strong concerns about food, the crucial role of eating, a link between diet and self-esteem, and they all have negative social and health consequences. Contrarily, ON seems to differ from EDs in focusing on food quality rather than food quantity [8]. OCD and ON appear to be similar regarding cognitive rigidity, perfectionism traits, obsessions, and compulsions related to healthy food. But where OCD obsessions are usually perceived as ego-dystonic and patients experience severe distress [10] ON obsessions are perceived as normal and adequate [8, 11].

Recent studies have made it clear that there is no proper distinction between the term “healthy orthorexia” and the pathological form of unhealthy eating, Orthorexia Nervosa (ON) [12, 13]. In 2004, the first peer-reviewed article about ON was published, in which a tool, ORTO-15, was presented to measure the disorder/syndrome [14]. Today, the ORTO-15 and the Orthorexia Self-Test (also known as the Bratman Orthorexia Test) [15] are the most commonly used ON instruments. This instrument has received a series of criticism in terms of the credibility of their findings. The ORTO-15 has been accused of overestimating the prevalence of ON, because it incorrectly identifies dieting as unhealthy/harmful, without investigating its pathology [16]. It might be more likely that ORTO-15 is good at identifying people who are serious about eating healthy, but not good at identifying those whose healthful dietary choices are associated with pathology [17]. Validity, reliability and internal consistency have been questioned as well [18]. The Orthorexia self‑test (BOT) developed by Bratman was the first questionnaire created to assess ON. The BOT was constructed based on characteristics of ON that Bratman identified in daily practice. Despite its clinical origin it is looked upon as a tool of limited clinical utility and seem to lack proper validation [18–20].

Ten years after the first peer-reviewed paper was published, Moroze and colleagues proposed the first diagnostic criteria [21]. Several definitions and diagnostic criteria for ON have since been proposed, but consensus has so far not been reached, and ON is yet to be included as a formally recognised disorder in any psychiatric diagnostic system [22]. Papers about its prevalence and its correlates as well as case studies seemed to make up a major proportion of the academic literature regarding ON [21, 23–25].

Today it is general knowledge that physical exercise and a healthy diet are important pieces in building an optimal health [26]. The focus on achieving an optimal health can however become obsessive and unhealthy, and some may in this process develop distress/symptoms [13, 27]. As eating disorders generally are well known in the sport and exercise population [28–32], it comes as no surprise that instances of ON are also linked to peoples’ physical activity level [33–36]. It is therefore of interest to investigate how widespread ON really is in the exercising population. We hypothesized that we would find high prevalences of ON in the exercise population. In this study, the term exercise is defined as any activity requiring physical effort, carried out to sustain or improve health and fitness. A recent meta-analysis showed a correlation of 0.12 between ON and exercise and a correlation of 0.29 between ON and exercise addiction [13]. Despite the fact that there is a link between exercise and ON /ON tendencies [28], prevalence studies of ON in sports and exercising populations present large discrepancies in terms of results [17].

In the same way that eating disorders in general have a higher incidence and prevalence in certain groups, it is conceivable that the same also applies to ON. Some studies shows that females are at higher risk of developing eating disorders than men [36]. There is some controversy and inconsistent findings when it comes to female preponderance regarding eating disorders [37–42]. Earlier literature does suggest that females are overrepresented among those suffering from eating disorders [43, 44]. To investigate the hypothesis that females are overrepresented among those with ON, we included females as one of the moderators in a meta-regression.

Because eating disorders are often linked to a distorted self-image [45], it is likely that those in question have a strong focus on themselves. Studies suggest that athletes in individual sports suffer more frequently from depression [46, 47], are more prone to feel the effects of anxiety on performance [47, 48], and are more engaged in the perfectionists behaviours [49] than those involved in team sports, probably because individual athletes are more concerned about goals, whereas team sport members are more motivated by having fun [47]. Further we may assume that athletes in teams sport suffer less from stress than those involved in individual sports. A rationale for the assumption may be found in general stress theory stating that the stress reaction depends on both the individual’s appraisal of the stressor and the expectation to the outcome of the situation [50]. If the individual expects to have enough resources either within themselves or in the environment to handle the stressor, the stress reaction will be short lasting and optimal for performance. It is therefore conceivable that team members are less vulnerable to stress, simple because there are more available coping resources in a team. In addition, the performance of individual athletes is more under scrutiny than that of team sport members [47, 51]. Based on the above, we assumed that ON occurs more frequently in individual sports or types of exercise than in team sports and exercises.

Although sample size probably affects estimates less often in prevalence studies than in trial studies, there is still some evidence to suggest that small study effects (tendency that small studies are associated with higher prevalences/effects than larger studies) may influence prevalence estimates [52]. Hence, sample size should be investigated as a potential moderator for prevalence estimates across ON studies.

As mentioned above, the prevalence of ON in exercising/sports samples seems to vary considerably. Hence, estimating an overall prevalence and elucidating factors associated with potential dispersion of prevalences can help advancing the research field and inform clinicians and practitioners.

So far, no systematic review or meta-analysis of the ON prevalence within this population has been conducted, although a meta-analysis can provide a quantitative synthesis of the individual findings as well as identify potential variables moderating the prevalence figures across studies. Against this backdrop, we conducted a meta-analysis of all prevalence studies of ON in exercising/sports samples. In addition, a meta-regression including sex, type of sport (individual, team, mixed/unknown), and sample size was conducted to identify potentially relevant moderators.

## Methods

### Protocol and guidelines

**Fig. 1:**
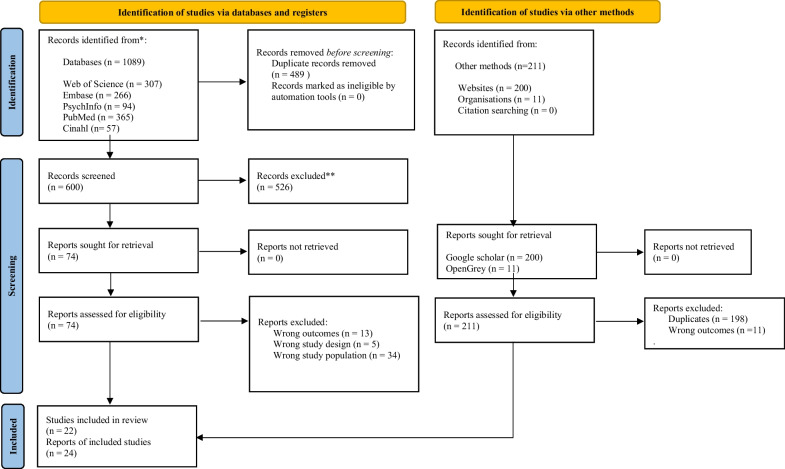
PRISMA 2020 [30] flow diagram for new systematic reviews for ON prevalence

This systematic review and meta-analysis were pre-registered with PROSPERO (CRD42022301749) and adhered to the guidelines found in the Preferred Reporting Items for Systematic Reviews and Meta-Analyses (PRISMA) procedure [53, 54] as well as the recommendations for the Meta-analysis of Observational Studies in Epidemiology (MOOSE) [55]. The first PROSPERO registration for this metanalysis only covered “athletes” as a sample, but it was changed to “exercising populations” due to the initial search producing extremely few studies. Figure 1 presents the literature search and selection process. See Appendix A for a completed Additional file 1: PRISMA-guideline checklist [54].

### Systematic search strategy

Systematic searches and a comprehensive literature search were conducted in five electronic databases—PubMed, Embase, Web of Science, PsychInfo, and CINAHL. In addition, we searched through Google Scholar and OpenNet in order to identify potential grey literature in the field. The literature searches were conducted between January 20, 2022, and February 1, 2022. A supplementary search was conducted September 10, 2022. The following keywords were used: Orthore* AND (prevalenc* OR incidenc* OR frequen* OR cut-off OR epidem*). Reference lists of included articles were further hand-searched for the identification of relevant articles for inclusion. No restrictions in terms of time frame were used. The search strategy and the keywords were approved by a librarian at the University of Bergen.

### Study selection criteria

The key inclusion criteria for the articles in this systematic review and meta-analysis were as follows. (1) The study informed about the prevalence of ON among athletes or people who exercise frequently. “Frequent exercisers” were defined as subjects who exercised at least once a week, and all types of physical activities/exercise and sports were included. Athletes were defined as subjects who should fulfilled four criteria; (1) training in sports aiming to improve his/her performance or results; (2) participating actively in sport competitions; (3) are formally registered in a local, regional or national sport federation as a competition;% and (4) have sport training and competition as his/her major activity or focus of interest, almost always devoting several hours in all or most of the days to these sport activities, exceeding the time allocated to other professional or leisure activities [56]. (2) The study presented original data on the prevalence of ON. (3) The study was published in a European language. (4) The study used a tool for measuring ON where the procedure provided a categorisation of pathological ON (e.g., based on cut-off scores or an interval that was categorised as either “ON”, “risk of ON”, or “tendencies of ON”. In the analysis, we used the most liberal cut-off scores (the scores that provided the highest prevalence) if the study reported more than one. This decision was based on the most liberal cut-off score for the ORTO-15 as recommended by the scale constructors [57].

The exclusion criteria were as follows: (1) participants stemming from a clinical sample only. We decided to exclude clinical samples because we wanted the samples to reflect the general exercising population; (2) studies reporting the prevalence of “healthy orthorexia”; (3) studies only reporting mean scores on ON measures, hence failing to report proportions/percentages scoring above cut-offs/categorisation of ON; and (4) studies based on qualitative data, case studies, interviews, case reports, or reviews. Two reviewers (SMH and JB) performed title/abstract and full-text screenings independently of each other. A third reviewer (SP) participated in the final discussion about the included articles. Discrepancies were resolved through discussions.

### Data extraction

The following study and participant characteristics were extracted from the identified studies and coded into a data extraction template, including author, year of publication, country, and continent, study type, sample type, type of selection, sex, age range (mean ± SD), age category, total sample size, percentage exercisers, percentage of women, frequency of exercising, type of ON measurement, cut-off/score-interval used for measurement of ON, type of sport (individual, team, mixed/unknown), prevalence of ON, and response rate.

In the categories “country and continent”, we stated the country/continent of the manuscript’s and participants’ origin. If several countries/continents were mentioned, we selected all that applied.

In the category “sample type”, we chose between the following four options: (1) general population, (2) students, (3) athletes/exercisers, and (4) others. A specific category was scored if 75% or more of the sample consisted of that category. If the sample consisted of several categories, then all the categories could be coded for the respective study. We defined the sample as athletes or exercisers if those in the sample were exercising at least once a week or fit the definition of athletes being used [56], that is, if the sample was defined as yoga practitioners, if the whole sample was selected from a fitness gym or a CrossFit-centre, or if the article authors specifically named the sample as athletes. If the study did not provide demographic information about the exercising population, we used information about the whole sample.

The age category contained five different age groups: (1) adolescents 15–18 years, (2) young adults 18–34 years, (3) adults 35–64 years, (4) older adults 65 + years, and (5) mixed ages. The sample ended up in one of the first four categories if 75% or more belonged to one specific age category. The category “type of selection” had two options: (1) random population sampling and (2) non-random sampling.

In the category “type of sport,” we distinguished between three codes. (1) Individual sport contained athletes/exercisers such as runners, dancers, gymnasts, boxers, wrestlers, martial artists, cyclists, figure skaters, Olympic weightlifters, powerlifters, bodybuilders, and CrossFitters. People belonging to a fitness gym or yoga center were also included in this category on the basis that they do not work together on a team to achieve a goal. (2) Team sport contained every sport where one performs as a team, e.g., football, hockey, rugby, netball, cheerleading, lacrosse, baseball, handball, basketball, or floorball. (3) Mixed/unknown was used if both individual and team sports athletes/exercisers were included or if the study did not inform about which type of sport/exercise the participants performed. The athletes/exercisers ended up in one of the two first categories if 75% or more of the sample consisted of that category.

The extraction was conducted independently by two reviewers (SMH and JB). Disagreements were resolved through discussions.

### Risk of bias assessment

All the included studies were assessed for risk of bias using a modified quality assessment checklist for population-based prevalence studies developed by Holy et al. [58]. This risk of bias assessment contains 10 items reflecting different characteristics of the included articles aiming at evaluating their internal and external validity. Each of the 10 items was scored as either “yes” (0-points, low risk of bias) or “no” (1-point, high risk of bias). A high risk of bias was indicated as follows: (1) the target population was not representative of the national population, (2) the sampling frame was not representative of the overall target population, (3) the sample was not randomly selected, (4) the response rate was less than 65%, (5) data were collected from a proxy, (6) no acceptable definition or delimitation of ON was used, (7) the ON measurement instrument was not shown to have reliability or validity, (8) the same mode of data collection was not used for all participants, (9) the shortest prevalence period for the parameter was not suitable, and (10) the numerator(s) or denominator(s) were not suitable. Based on the 10 items, a composite risk of bias score was calculated: high risk of bias (7–10 points), moderate risk of bias (4–6 points), or low risk of bias (0–3 points). Two reviewers (SMH and JB) conducted the risk of bias assessment independently of each other, and in case of disagreement, consensus was reached through discussions. The results are present in Table 1.

**Table 1:** Risk of bias/methodological quality [36] of included studies

Study ID	1. N-representativeness	2. N-frame	3. Random selection	4. Non-response bias	5. Primary data	6. Operationalization	7. Instrument	8. Consistency	9. Period	10. Estimation	Total Risk^§^ score	Risk Category
Almeida et al. [34]	1	1	1	0	0	0	0	0	0	0	3	Low
Bert et al. [76]	1	1	1	0	0	0	0	0	0	1	4	Moderate
Bo et al. [75]	1	1	1	0	0	0	0	0	0	0	3	Low
Civil [79]	1	1	1	0	0	0	0	0	0	0	3	Low
Clifford and Blyth [4]	0	1	1	0	0	0	0	0	0	0	2	Low
Dabal [80]	0	1	1	0	0	0	0	0	0	0	2	Low
Dunn et al. [17]	1	1	1	0	0	0	0	0	0	0	3	Low
Erkin and Göl [78]	1	1	1	0	0	0	0	0	0	0	3	Low
Freire et al. [77]	1	1	1	0	0	0	0	0	0	0	3	Low
Kattan [84]6	1	0	0	0	0	0	0	0	0	0	1	Low
Keller and Konradsen [71]	1	1	1	0	0	0	0	0	0	0	3	Low
Kiss-Leizer et al. [83]	1	1	1	0	0	1	0	0	0	0	4	Moderate
Labossiere and Thibault [86]	1	1	1	1	0	0	0	0	0	0	4	Moderate
Lewis [70]	1	1	1	1	0	0	0	0	0	0	4	Moderate
Malmborg et al. [82]	0	1	1	1	0	0	0	0	0	0	3	Low
De Marchi and Baratto [68]	1	1	1	1	0	0	0	0	0	0	4	Moderate
Rizzieri et al. [74]	0	1	1	0	0	0	0	0	0	0	2	Low
Rudolph and Göring [36]	0	1	1	0	0	0	0	0	0	0	2	Low
Segura-Garcia et al. [33]	1	1	1	0	0	0	0	0	0	0	3	Low
da Silva et al. [72]	1	1	1	0	0	1	0	0	0	0	4	Moderate
Surala et al. [16]	0	1	1	0	0	0	0	0	0	0	2	Low
Tocchetto et al. [73]	1	1	1	0	0	0	0	0	0	0	3	Low
Uriegas et al. [69]	1	1	1	0	0	0	0	0	0	0	3	Low
Herranz Valera et al. [85]	1	1	0	1	0	0	0	0	0	0	3	Low

Item score: (0: low risk, 1: high risk). ^§^Total quality/risk score: [range (0–10): high quality/low risk (0–3), moderate quality/risk (4–6), poor quality/high risk (7–10)]

### Data synthesis and analysis

For all types of coding (inclusion/exclusion, study characteristics extraction, and risk of bias evaluation), percentages of the initial agreement between the two reviewers were calculated. The prevalences and the corresponding 95% confidence intervals (95% CIs) of the included studies were synthesised using a random-effects model, which did not assume that the included studies came from the same population of studies [59]. The between-study variance was estimated by using the Der Simonian and Laird approach [60]. Heterogeneity was assessed based on Cochran’s *Q* and *I*^2^ statistics, the latter of which reflects the proportion of variation in the observed effects that is due to variation in true effects [61]. According to Higgings et al. [62], an *I*^2^ of 0% suggests no heterogeneity, 25% suggests low heterogeneity, 50% suggests medium heterogeneity, and 75% suggests high heterogeneity. Publication bias was investigated by the Egger test [63] and Duwal and Tweedie’s trim and fill procedure. The latter is based on a funnel plot with the largest and most precise studies situated at the top (y-axis) of the funnel plot with the effect size situated along the x-axis. The trim-and-fill procedure trims off asymmetric outlying studies and replaces them with studies around the center, whereupon an adjusted effect size and 95% CI are calculated [64]. A random-effects meta-regression analysis was conducted to examine whether the following predictors explained heterogeneity in ON prevalence: (a) percentage women, (b) type of sport (1 = individual, 2 = team, and 3 = mixed/unknown, where the latter category comprised the reference), and c) sample size. In addition, a sub-group analysis based on the tool used to assess ON symptoms (ORTO-15; k = 18, ORTO-11; k = 3, and others; k = 3) was conducted. The subgroup analysis was based on the recommended [65] mixed effects model consisting of a random-effects model within subgroups pooling tau across groups as well as a fixed-effect model across subgroups. A sensitivity analysis was conducted only including studies based on non-student samples. The meta-analysis and the meta-regression analysis were conducted using the Comprehensive Meta-Analysis 3.0 software [66]. When calculating the prevalences, the software logit transforms the prevalences in order to correctly carry out all of the statistical analyses, before they are back-transformed to the metric of the prevalences.

## Results

### Literature search

A total of 1089 hits were identified from databases and registers (Web of Science, Embase, PsychInfo, PubMed, and CINAHL), and 211 hits were identified from other methods/grey literature (Google scholar and Open Grey). See flow chart Fig. 1. A total of 22 studies were finally included from databases and registers whereas two were identified through identification of studies via other methods, amounting to a total of 24 studies being included.

The inter-rater reliability of the study screening procedure was provided by the Covidence software and showed a proportional agreement of 96.0% for the title/abstract screening and 94.7% for the full-text screening. For some parameters, there were substantial disagreements which not necessarily reflects a problem [67] but rather spurs discussion, finally resulting in a consensus being reached.

### Description of the studies

The studies included a total of 7592 participants, ranging from 41 [68] to 1090 [69] respondents with a mean of 316 (SD = 3) participants. In total 4288 participants were females, and 3304 were males. Table 2 presents further characteristics of the included studies.

**Table 2:** Characteristics of prevalence studies of exercise populations

Study ID	Country	Selection	Sample type	Age range	Age Mean ± SD	ON instrument	Cut-off	N	% Female	Sport type	ON prev%	RR %
Almeida et al. [34]	Portugal	Non-random	Exercisers	18–59	32.8 ± 11.6	ORTO-15	35	193	58.5	Individual	51.8	100
Bert et al. [76]	Italy	Non-random	Athletes	18–40	26.5 ± 5.4	ORTO-15	40	367	25.5	Mixed/unknown	71.9	100
Bo et al. [75]	Italy	Non-random	Students	Not provided	19.9 ± 1.8	ORTO-15	35	200	32.5	Mixed/unknown	26.5	87.0
Civil [79]	Turkey	Non-random	Athletes	18–37	22.9 ± 4.3	ORTO-11	27	143	31.5	Individual	75.5	100
Clifford and Blyth [4]	UK	Non-random	Students & athletes	18–27	21 ± 1.0	ORTO-15	40	116	58.0	Team sport	76.6	100
Dabal [80]	Poland	Non-random	Students	18–35	23.9 ± 4.7	ORTO-15	35	83	49.6	Mixed/unknown	37.3	100
Dunn et al. [17]	USA	Non-random	Students	Not sited	21.7 ± 4.8	ORTO-15	40	264	68.0	Individual	74.2	93.5
Erkin and Göl [78]	Turkey	Non-random	Athletes	18–56	30.5 ± 9.2	ORTO-11	27	118	92.4	Individual	75.0	77.1
Freire et al. [77]	Brazil	Non-random	Exercisers	18–34	26.6 ± 7.8	ORTO-15	40	60	63.3	Individual	80.0	100
Kattan [84]	Lebanon	Random	Students	18–30	21.7 ± 1.9	ORTO-15	40	152	84.0	Mixed/unknown	68.4	100
Keller and Konradsen [71]	Denmark	Non-random	Exercisers	16–29	22 ± 3.4	OS	7	119	53.8	Individual	8.4	100
Kiss-Leizer et al. [83]	Hungary	Non-random	General population	18–72	29.7 ± 10.2	ORTO-11	14–19	635	79.2	Individual	36.9	100
Labossiere and Thibault [86]	Canada	Non-random	Students & athletes	Not provided	21.3 ± 1.7	ORTO-15	35	133	71.4	Mixed/unknown	11.3	31.6
Lewis [70]	USA	Non-random	Students & athletes	18–23	20.5 ± 1.2	ORTO-15	40	427	44.0	Team sport	66.3	71.0
Malmborg et al. [82]	Sweden	Non-random	Students	19–29	22.8 ± 2.2	ORTO-15	40	118	54.2	Mixed/unknown	78.8	66.1
De Marchi and Baratto [68]	Portugal	Non-random	Students	18–39	21 ± not provided	ORTO-15	40	41	93.9	Mixed/unknown	73.2	96.5
Rizzieri et al. [74]	Portugal	Non-random	Exercisers	20–59	29.9 ± 7	ORTO-15	40	65	55.4	Individual	89.2	100
Rudolph and Göring [36]	Germany	Non-random	Athletes	Not provided	29.4 ± 11.6	DOS	30	1008	44.5	Individual	4.3	100
Segura-Garcia et al. [33]	Italy	Non-random	Athletes	16–45	22.3 ± 4.7	ORTO-15	35	577	32.8	Mixed/unknown	28.8	86.8
da Silva et al. [72]	Brazil	Non-random	Exercisers	18–60	27.8 ± 5.1	TOS	50–75	226	36.3	Individual	5.3	100
Surala et al. [16]	Poland	Non-random	Athletes	14–39	20.9 ± 4.7	ORTO-15	40	273	45.8	Mixed/unknown	88.3	100
Tocchetto et al. [73]	Portugal	Non-random	Students & athletes	Not sited	23.5 ± 1.4	ORTO-15	40	50	46.0	Mixed/unknown	78	100
Uriegas et al. [69]	USA	Non-random	Students & athletes	18–40	19.6 ± 1.4	ORTO-15	40	1090	69.4	Mixed/unknown	67.9	98.6
Herranz Valera et al. [85]	Switzerland	Non-random	Athletes	20–55	37 ± 6.7	ORTO-15	40	136	65.5	Individual	86.6	23.4

*ON* othorexia nervosa, *Prev* prevalence, *RR* response rate, *OS* orthorexia screen, *DOS* Düsseldorfer Orthorexie Scale, *TOS* Teruel Orthorexia Scale

Cut-off, a mark for the lowest point at which ON is attained. Sample type, divided in which groups total samples is recruited from (75% or more)

Of the 24 included studies, publication years ranged from 2012 [70, 71] to 2021 [69, 72]. Studies were conducted in Portugal (*k* = 4: [34, 68, 73, 74]), Italy (*k* = 3: [33, 75, 76]), the US (*k* = 3: [17, 69, 70]), Brazil (k = 2: [72, 77]), Turkey (k = 2: [78, 79]), Poland (k = 2: [16, 80]), and one study each from the UK [4], Germany [81], Sweden [82], Denmark [71], Hungary [83], Lebanon [84], Spain [85], and Canada [86].

Samples were mostly recruited from fitness gyms, sports universities, or specific groups of athletes (k = 17: [4, 16, 33, 34, 69–74, 76–79, 81, 85, 86]). For six studies the sample was recruited from regular universities (students) [17, 68, 75, 80, 82, 84], and one study was based on a general population sample [83]. All of the studies had cross-sectional study designs and used a non-random selection method—except for one study that employed a random selection of participants [84]. All of the studies were peer-reviewed research papers, except for two studies that were theses (one master thesis [84], one doctoral thesis [70] and one study published in a journal for Danish nurses [71].

The majority of studies (k = 18: [4, 16, 17, 33, 34, 68–70, 73–77, 80, 82, 84–86]) assessed ON using the ORTO-15 questionnaire [14]. In addition, three studies [78, 79, 83] used the ORTO-11 questionnaire [87], one study [71] used the Orthorexia Screen [88], one study [81] used the Düsseldorf Orthorexia Scale [89], and one study [72] used the Teruel Orthorexia Scale [90].

### Prevalence estimates and heterogeneity

**Fig. 2:**
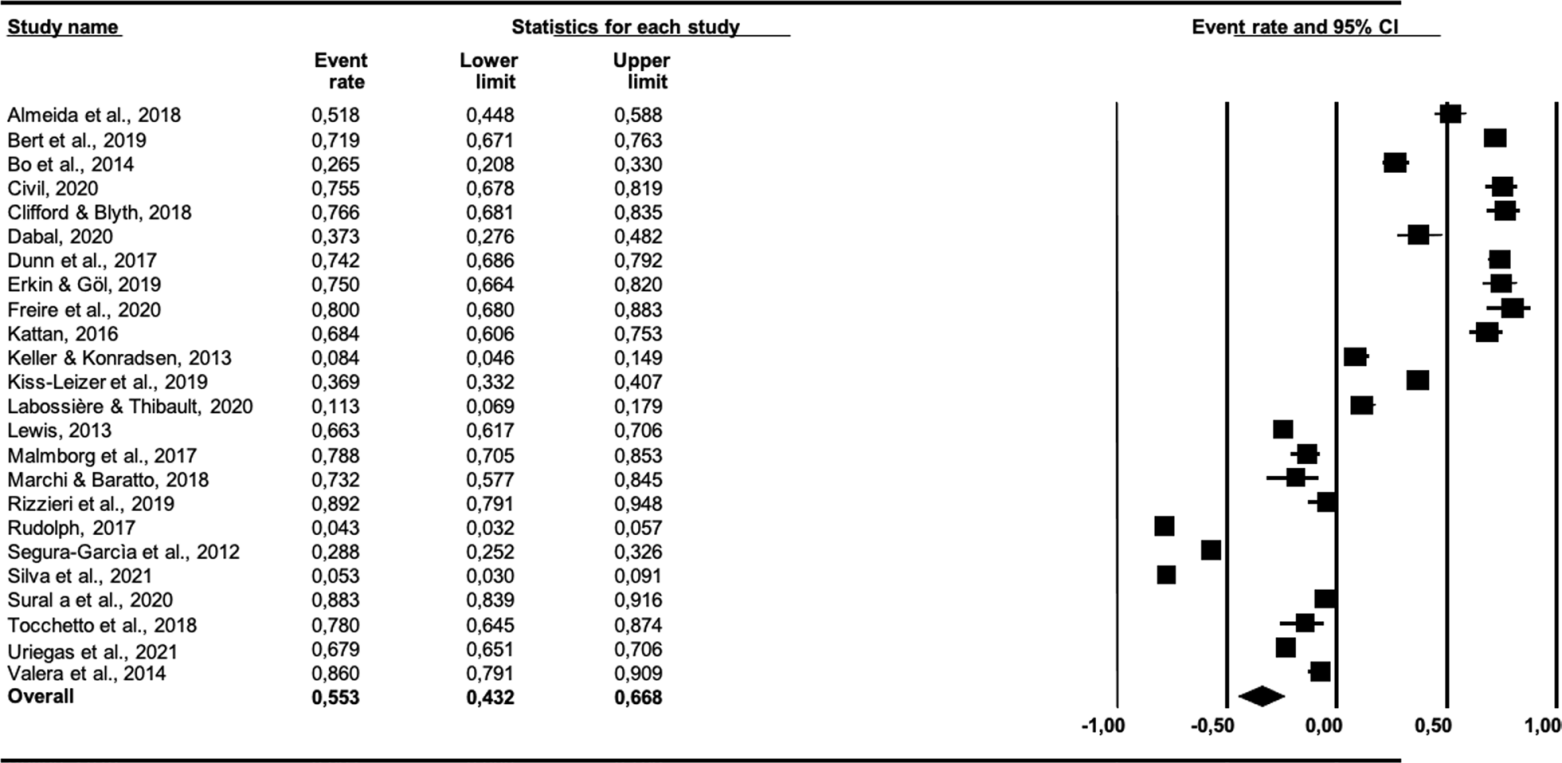
Forest plot of the included studies

The results of the meta-analysis are presented as a forest plot (Fig. 2). The overall prevalence across all 24 studies was 55.3% (95% CI 43.2–66.8). Cochran’s *Q* was significant (*Q* = 1,436.38, *df* = 23, *p* < 0.0000), suggesting heterogeneity across the prevalence estimates, and the *I*^2^ statistic was 98.4%, indicating very high heterogeneity.

### Correlates of ON prevalence

Because of the significant heterogeneity, a meta-regression analysis based on a random-effects model was conducted including the percentage of females, type of sport (individual = 0, team = 1, mixed/unknown = 2), and sample size as predictors. The results are present in Table 3. Overall, the regression model was not significant (*Q* = 5.97, *df* = 4, *p* = 0.2011, pseudo *R*^2^ = 0%).

**Table 3:** Results of meta regression of percentage female, type of sport and sample size on ON prevalence among the exercising population

Predictor	Coefficient	SE	95% CI	Z-value	2-sided *p*
Percentage female	0.0176	0.0145	− 0.0109, 0.0460	1.21	0.226
Indvidual sport^a^	− 0.4420	0.5602	− 1.5000, 0.6559	− 0.79	0.430
Team sport^a^	0.6575	0.9990	− 1.3006, 2.6155	0.66	0.511
Sample size	− 0.0016	0.0010	− 0.0035, 0.0003	− 1.71	0.087

*SE* standard error, *95% CI* 95% confidence interval

^a^Mixed sports/unknown comprised the reference category

Percentage of females (*b* = 0.018, *p* = 0.23), individual sport and team sport (*b* = – 0.442, *p* = 0.43, *b* = 0.658, *p* = 0.511; mixed/unknown comprised the reference), and sample size (*b* = – 0.002, *p* = 0.09) were accordingly unrelated to ON prevalence among the exercise populations. It should be noted that lack of significant associations does not necessarily imply negative findings as several studies lacked key information and was consequently coded as “unknown”.

### Publication bias

**Fig. 3:**
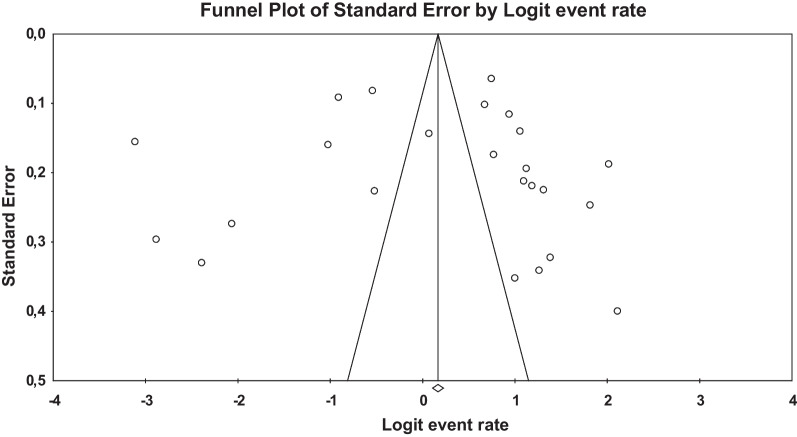
Funnel plot of the included studies

The results of the Egger test (*b* = 0.312, 95% CI = –6,891 to 7.52, *t* = 0.090, *p* 1-tailed = 0.465, *p* 2-tailed = 0.929) did not suggest publication bias. The funnel plot (Fig. 3) suggested a minor deviance from symmetric distribution, thus suggesting a lack of studies to the left of the distribution. The Duwal and Tweedies’ trim-and-fill procedure trimmed three studies and consequently implied a minor change in the overall estimated prevalence (51.4%, 95% CI 39.6–63.9%).

### Subgroup analysis

The subgroup analysis was significant (Q_bet_ = 33.6, *df* = 2, *p* < 0.01) and showed high prevalences associated with ORTO-15 (65.6%, 95% CI 55.1–74.7%) and ORTO-11 (63.4%, 95% CI 37.4–83.4%), but far lower prevalences associated with the other instruments (5.7%, 95% CI 0.2.1–15.2%).

### Sensitivity analysis

Results from the sensitivity analysis including only studies (k = 13) based on non-students samples showed an overall prevalence of 51.3% (95% CI 32.3–70.0).

## Discussion

A total of 24 studies fulfilled the inclusion criteria and were consequently included in the meta-analysis. The studies yielded an overall ON prevalence of 55.3%. The dispersion of effect sizes was significant, ranging from 4.3% [81] to 89.2% [74]. The prevalence of ON was overall very high across the included studies and suggests that approximately over half of the population is suffering from it, indicating that ON tendencies might be a frequent phenomenon among exercising populations. Hence, a significant proportion of those who aim to achieve and/or maintain good physical health may be prone to a preoccupation with healthy eating to a dysfunctional level [16, 33, 73, 79, 81, 83]. Accordingly, focusing on the prevention and treatment of ON among exercising populations should be prioritised [91].

The reported prevalence of ON was considerably higher compared to the prevalence of eating disorders in general among athletes, which is estimated to be 13.1% [29], but is still comparable to the ON prevalence in the general population (41% estimated by the ORTO-15) [88], and the small correlation found between ON and exercisers [13].

Although these findings can be regarded as support for the high prevalence of ON in exercising populations, the findings should still be interpreted with caution. ON is currently not acknowledged as a diagnostic entity in formal psychiatric nosology (ICD-11 and DSM-V), and thus there is still considerable discussion about the diagnostic criteria, methods and tools used for its classification [88]. Furthermore, pathological ON often overlaps with healthy orthorexia and orthorexic behaviors [92]. In that way, assessment of ON may sometimes provide false positive results as people concerned with healthy eating may be over-pathologized as ON sufferers.

Accordingly, many have criticised the methods used to assess ON as too unspecific and insensitive, having various cut-offs, and based on different definitions of ON [5, 21, 92–94]. Also, to what extent ON represent at the conceptual level represent a pathological state or rather a sensible approach to healthy eating is debated [12]. Still, it cannot be ruled out that the high prevalence reflects real problems, mirroring the more invisible symptoms and characteristics of ON in contrast to the more tangible symptoms associated with other eating disorders such as anorexia nervosa and binge-eating disorder [95, 96]. In addition, the high prevalence of ON can also reflect a willingness to report, and even over-report, the relevant symptoms, which in contrast to other eating disorders may be regarded as desirable, which seems to be the case for symptoms of exercise addiction [97].

In all, 11 of the 24 studies consisted of students, where the mean age across those studies was 18.8 years [4, 17, 68–70, 73, 75, 80, 82, 84, 86]. This may also have an impact on the prevalence. Another factor that may affect the prevalence is geographical location. Only seven [17, 69, 70, 72, 77, 84, 86] of the included studies were conducted outside Europe, and only three [72, 77, 84] of these stemmed from non-western countries. The predominance of studies from western countries may be yet another reason for the high prevalence rates because studies on other eating disorders (e.g., anorexia nervosa) show a higher incidence in western countries compared to non-western countries [39, 88].

The high heterogeneity in terms of prevalence rates of ON found in this review resonates well with the differences in ON prevalence reported in the general population, where it varies between 6.9 and 75.2% [88]. This large disparity in prevalence might be explained by some of the same factors mentioned above; however, it may also reflect divergence from other study characteristics as well. In order to elucidate this further in terms of the present study, a meta-regression analysis with three independent variables was conducted. The independent variables included sex (percentage females), type of sport (individual, team sport, mixed/unknown), and sample size.

However, none of the independent variables were significantly associated with the prevalence rates. This implies that other study characteristics may explain the heterogeneity. Possible candidate variables in this context are risk of bias and other sample characteristics. It is further conceivable that athletes compared to more recreational exercisers put stronger emphasis on diet and thus would score higher on orthorexic tendencies. Other potential moderators entail age [98], student status [99], status as vegetarian/vegan [100], psychiatric comorbidity [101], and level of physical activity [102]. These should thus be investigated in future studies. Pre-registration and a reasonable ratio of moderators to number of effect sizes put restrictions on how many moderators that it would be appropriate to include. Still, it should be noted that the sub-group analysis did turn out significant and showed that far higher prevalences were associated with the ORTO-15 and ORTO-11, compared to the other instruments. This shows that prevalence estimates may be heavily dependent on the instrument used for the assessment of ON and may suggest that administration of some of the most commonly used instrument assessing ON may result in a significant overestimation of the prevalences. The sensitivity analysis excluding all studies solely or partly based on student samples supported the overall high prevalence rate of ON reported in the present meta-analysis, probably because the studies retained relied heavily on ORTO-15 and ORTO-11.

As orthorexic tendencies are clearly a widespread phenomenon, people who work with students, athletes, elite sports, and other presumed risk groups (such as teachers, coaches and sports psychologists) should be aware of these tendencies [103–105]. In this way, one may prevent subjects at risk from turning from healthy orthorexia to obsessive/pathological orthorexia. This might be done by psychoeducation about ON and administering tools to disclose this. In terms of assessment the focus should also be on developing validated tools with good psychometric properties suitable to measure and capture these tendencies [18]. As mild mental problems may instigate development of more serious problems, early detection of ON tendencies is important [106].

### Limitations of the included studies

Notably, one fourth of the included studies had a moderate risk of bias, typically associated with low external validity. Further, it should be noted that only one of the included studies used random selection to recruit the sample [84]. Two of the included studies were not published in a peer-reviewed journal [70, 84], and two of the included studies did not use a specific cut-off and rather used a score-interval that resulted in a “high risk” in terms of internal validity regarding defining ON [72, 83]. Hence, future studies should improve especially concerning these study dimensions in order to move the field forward. Further, it is conspicuous that all studies were based on a cross-sectional design. Some studies had limitations in terms of reporting, since in eight studies only the demographic information of the total sample was reported, but nothing specific to those included as exercisers [17, 68, 75, 80, 82–84].

### Limitations and strengths of the present meta-analysis

Overall, the high study heterogeneity in terms of prevalence estimates, the moderate risk of bias in 25% of the studies, issues associated with the measures of ON and paucity of reporting of some key information in the included studies imply that the findings of the present meta-analysis should be interpreted by caution. Almost half [16, 33, 68, 69, 73, 75, 76, 80, 82, 84, 86] of the included studies lacked information about the type of sport and were consequently grouped as unknown for this parameter. In addition, detailed demographic information about the exercising sub-samples was sometimes lacking, hence descriptive data for the whole samples was in these cases included in the analysis. This represents limitations regarding sex and type of sport as moderators. Still, it should be noted that the authors of the present meta-analysis contacted the study authors in an attempt to obtain missing information. The broad definition of a “frequent exerciser” (at least once a week) could represent a too lenient definition of exercisers and might as such influence the findings. The present meta-analysis targeted the inclusion of grey literature, as recommended for the calculation of non-biased estimates in meta-analyses [59]. Still, the inclusion of grey literature is debatable. Some suggest that it makes meta-analyses more complete [65] whereas other argue that unpublished data are unlikely to have a significant impact on findings in meta-analyses [107], that unpublished studies may be of lower methodological quality than published studies [108] and that retrievable unpublished papers are not representative of unpublished literature in general [109]. No restriction in terms of time frame was applied, and articles in all European languages were included. Moreover, the meta-analysis was conducted in line with the updated Additional file 1: PRISMA guidelines [53]; these all are strengths of the present study. Although searches were conducted in several relevant databases, we cannot rule out that some relevant papers were excluded. All prevalence data and quality assessments of the included studies were coded independently by two reviewers. A limitation is that, although two reviewers coded inclusion/exclusion, extraction of data from included papers and “risk of bias”-inter-rater reliability was only available for the study screening procedure. Some of the included articles had several cut-offs, of which the most liberal was selected, which to some degree may have inflated some of the single as well as the overall prevalence estimates. Finally, it should be noted that cut-off scores of ON-related instruments are often not validated in the countries where they are used.

## Conclusions and recommendations for further studies

The present meta-analysis revealed a high prevalence of ON, albeit with a large disparity between studies. Neither sex, type of sport, nor sample size explained this heterogeneity, but the most commonly used ON-instruments are associated with high prevalences compared to other instruments. To expand our knowledge in this field, we need a better definition and agreed-upon diagnostic criteria of ON. Sensitive and reliable measurements of ON should also be developed. In addition, more longitudinal studies are warranted in order to identify predictors and not just correlates of ON. Prevalence studies based on representative samples including respondents from multiple sports and non-western countries with wider age-ranges will also help advance the field. More knowledge of actual ON prevalence and predictors are instrumental in order to estimate the actual need for treatment and to develop targeted preventive efforts.

## Supplementary Information

**Additional file 1.** PRISMA checklist.

## Data Availability

The data are available in the selected studies. The dataset that was generated after extracting the themes from the selected studies is available upon a substantiated request to the corresponding author.

## Abbreviations

CI: Confidence interval
df: Degrees of freedom
*I*2: Heterogeneity statistics
ON: Orthorexia nervosa
ORTO-15: Orthorexia Nervosa 15 Questionnaire
SD: Standard deviation
